# COVID-19: Moral and Ethical Implications for Orthopaedic Spine Surgeons

**DOI:** 10.1155/2021/6682705

**Published:** 2021-07-15

**Authors:** Cris J. Min, Cesar Iturriaga, Victoria Wang, Rohit Verma

**Affiliations:** ^1^Northwell Health Plainview Hospital, Department of Orthopaedic Surgery, Plainview, NY, USA; ^2^Northwell Health Huntington Hospital, Department of Orthopaedic Surgery, Huntington, NY, USA; ^3^Donald and Barbara Zucker School of Medicine at Hofstra, Hempstead, NY, USA; ^4^University of Connecticut, Storrs, CT, USA; ^5^Northwell Health North Shore University Hospital, Department of Orthopaedic Surgery, Manhasset, NY, USA

## Abstract

The rapid spread of COVID-19 has made a significant impact on healthcare systems worldwide, with a large influx of patients prompting the cancellation of elective surgery in order to conserve resources and prevent the risk of exposure to the novel virus. In this case report, we present a 66-year-old male patient, with a history of cerebral palsy and developmental disabilities, exhibiting an increasing loss of function over the course of 10 days amid the COVID-19 pandemic. The patient was initially refused transport to the hospital by emergency medical services and later transported per independent request from his surgeon. Upon admittance to the hospital, the patient was found to have severe spinal cord compression with myelopathic symptoms and underwent an anterior cervical discectomy and fusion. This case highlights the need for more specific guidelines regarding the evaluation of a spinal injury by EMS and the hospital system amid a national crisis.

## 1. Introduction

In December 2019, the first case of coronavirus disease 2019 (COVID-19) caused by the severe acute respiratory syndrome coronavirus 2 (SARS-CoV-2) was documented in Wuhan, China [1]. Since then, COVID-19 has evolved into a global pandemic, affecting over 12 million people as of July 2020 [2]. The large population of affected individuals has put a strain on the global healthcare system. Personal protective equipment, operating rooms, and ventilators are in short supply throughout areas with large numbers of COVID-19 cases. In an effort to conserve resources and reduce spread of the virus, both the ethics and frameworks of our healthcare system have undergone a remodeling.

Amid the pandemic, spine surgeons faced multiple sources of moral distress caused by major changes in healthcare. One such source stemmed from physicians and hospitals recognizing a needed shift away from patient-centered healthcare towards public health-centered care. In order to conserve equipment and space for COVID-19 patients, surgeons were forced to make decisions that would benefit the public's health, rather than benefit a sole patient. This resulted in a reduced ability for a surgeon to respect a patient's choices [3]. Additionally, many hospitals had set out a mandate for surgeons to remain at home during the pandemic unless they were specifically called upon. This resulted in another source of moral distress, as surgeons were not able to provide care in the midst of a public health crisis [3].

Although telehealth was implemented in the place of some in-person visits, not all patients could be assessed in this manner. Patients that needed sutures removed for example or patients that required a more hands-on evaluation by the attending surgeon would still stand to benefit most from in-person care [4]. In this particular case, the use of telemedicine aided in Dr. Verma's assessment of the patient's case as emergent, leading to the patient's prioritization and rapid hospitalization following the telehealth visit. In an emergent case, as described in this case report, teleconsultation can be beneficial in the delivery of timely medical intervention and avoidance of debilitating, permanent consequences. In the midst of a pandemic, telehealth allows the surgeon to determine a baseline evaluation of the degree of urgency regarding a patient's case and the avoidance of unnecessary hospital visits in less emergent cases. At the same time; however, a patient may need to come in for a more accurate evaluation, and a remote visit can be offered as a precursor to determine whether a patient can be managed via telehealth versus whether additional imaging or an in-person visit is necessary [4].

Orthopaedic spine surgeons were additionally facing the mandated cancellation of elective surgeries in order to reduce the strain on hospitals, conserve resources, and slow down the spread of the virus. Due to these restrictions, spine surgeons were put in a position in which they needed to make difficult decisions regarding which patients were in most urgent need of surgery and which patient's surgical needs could be delayed. These decisions can be made more difficult in instances where postponing surgical spine treatment can lead to poorer recovery or irreversible neurological damage. Risk factors such as the contraction of COVID-19 postoperatively and the possibility of this delaying recovery need to be taken into account; minimizing the risk of a patient contracting COVID-19 in a hospital setting, namely, elderly patients who are most at risk for severe disease or death, should be considered by the surgeon [5]. Many hospitals have been developing protocols that stratify the urgency of specific cases. Conditions exhibited by the patient that may denote neurological compromise, such as new or progressing weakness, myelopathy from degenerative disease, and severe pain due to nerve compression, have been considered in need of urgent surgical intervention [6]. Additionally, patients at risk for permanent neurological damage have been deemed urgent cases [6].

Typically, orthopaedic surgeries are categorized into urgent inpatient, urgent outpatient, elective inpatient, and elective outpatient categories [7]. However, the COVID-19 pandemic and restriction of elective surgeries have resulted in a need for a more specific system to categorize cases based on the priority level.

Although there has not yet been a multi-institutional system implemented, a scoring system aimed at categorizing patient cases based on urgency has been developed by 16 spinal surgeons across the United States [8]. On a scale from -19 to 91, cases are categorized by the degree of progression and impairment caused by their deficit, with higher numbers corresponding to increasing urgency [8]. Patients that are at greater risk of postoperative complications, more severe neurological deficits, and spinal instability are additionally scored higher [8]. This scoring system may provide a guide to help in the prioritization of patients during COVID-19; however, the ultimate decision will still be left to the surgeon.

There are also guidelines formulated by the French Spine Surgery Society regarding the management of patients and urgency of surgery [9]. This is based on three levels of urgency: urgent surgical indications, surgical indications associated with a potential loss of chance for the patient, and nonurgent surgical indications [9]. Patients categorized in level 1 urgent surgical indication would require a procedure; however, patients categorized in levels 2 and 3 will have either a scheduled surgery with reasonable delay or a completely postponed surgery [9]. These models can aid in a surgeon's decisions on how to rank the urgency of surgeries.

New York State's largest healthcare provider, Northwell Health, uses similar guidelines. At Northwell, surgeons identified their cases from levels 0-4, with level 4 representing emergent surgeries and level 3 representing urgent surgeries, both of which were prioritized for surgery, whereas levels 2 and lower were postponed [10]. Level 0 indicates an elective surgery, where the timing of surgical intervention would not impact a patient's outcome. These classifications for prioritizing surgery are determined based on how harmful a time-based delay in surgical intervention would be for the patient. Cases that are deemed a level 4 priority indicate that the patient would be at an immediate risk for permanent disability or severe harm without surgery and indicate a need for immediate surgical intervention [10]. An accepted treatment protocol in the United States for spinal cord injury, which would render a case emergent, follows a suggested 8-24-hour surgical intervention time window for the best recovery results [5]. A level 3 case indicates that a patient has failed previous medical management of their condition and would face a prolonged stay/increased chance of another hospital admission if a delay in surgery occurred. Cases deemed urgent do not currently have a clear exact recommended timing of surgical intervention in the United States, although these cases are considered in need of prompt intervention, more so than levels 0-2 but not as immediate as level 1 cases [5]. Finally, level 2 surgeries are considered semiurgent, where a delay past three months would put the patient at a risk of harm, and a case considered level 1 indicates that a delay past six months would not put a patient at risk of harm [10]. The pause of elective surgeries in New York lasted from March 32rd, 2020, to June 28^th^, 2020 [11]. During this time, there were approximately 16,000 surgical cases postponed, with about 1,800 of these cases being orthopaedic, in order to conserve resources during the pandemic [10].

It was up to the surgeon to identify level 3 and 4 cases that were postponed and submit case information to their respective department chair to approve scheduling in the operating room. Then, the Surgical Emergency Operations Committee reviewed all data and provided oversight for all scheduled cases [10]. Once the case was approved, the surgery was scheduled and the patient underwent medical clearance exams and COVID testing. All COVID-positive patients were postponed, unless classified as level 4 [10].

Research done in Germany has revealed that restrictions on elective surgery have led to postponements affecting over 60% of patients that would otherwise be receiving surgery [12]. Once elective surgeries resume, these delays may lead to increased wait times in receiving treatment as well as higher readmission rates, as the delay in surgery may lead to a higher complication rate [13]. Delays in surgery, namely, anterior cervical discectomy and fusion surgeries, have been associated with a five-fold increase in hospital stay time and mortality rate as well as a higher rate of returning to the OR [14].

## 2. Case Report

A 66-year-old male with a past medical history of cerebral palsy, seizure disorder, and mental retardation with developmental disabilities presented to the North Shore University Hospital emergency department with symptoms of neurologic deterioration. At baseline, the patient was ambulatory with a walker with good functional status in upper and lower extremities.

Prior to admission, the patient's sister and healthcare proxy had contacted our attending orthopaedic spine surgeon, who was informed that 10 days prior the patient experienced multiple falls and progressive weakness, resulting in minimal ambulation. The patient had increasing loss of function of bilateral upper extremities, with right greater than left symptoms, and bilateral lower extremities and essentially became bed bound in the span of 10 days. The patient also experienced progressive difficulty with feeding himself.

Emergency medical services (EMS) had refused initial requests to acquire and transport the patient to the hospital due to concerns of safety and exposure during the COVID-19 pandemic. As a result, the attending surgeon independently contacted EMS and persuaded them to bring the patient to North Shore University Hospital for urgent evaluation and operative intervention.

Clinical examination of the bilateral upper extremities revealed 3/5 motor strength in the deltoids, biceps, and triceps, proximally and distally, and 1-2/5 strength in grip strength. Examination of bilateral lower extremities revealed 3-4/5 motor strength proximally in the hip flexors and knee extensors. Tibialis anterior, extensor hallucis longus, and peroneals were found to be 1-2/5 in motor strength. There was also reduced sensation throughout, in both the upper and lower extremities. The patient also had a positive Babinski sign, as well as positive Hoffman's sign. Increased deep tendon reflexes were also present. The patient did not exhibit any bowel or bladder dysfunction; however, given the signs and symptoms, the patient was given a modified Japanese Orthopaedic Association (mJOA) score of 8, defining the severity of myelopathy as severe [15].

**Figure 1:**
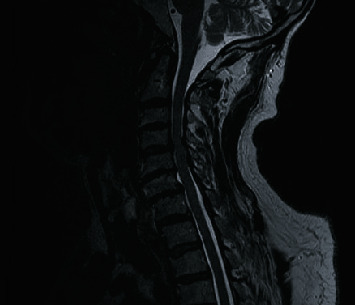
Sagittal T2-weighted MRI of the cervical spine, demonstrating spinal cord compression at the level of C4-C5.

**Figure 2:**
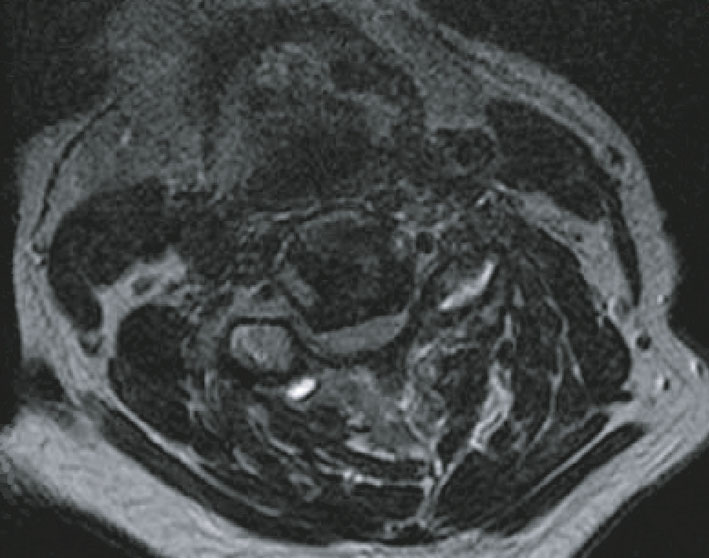
Axial T2-weighted MRI of the cervical spine, demonstrating severe canal stenosis with cord compression at the level of C4-C5.

MRIs of the cervical, thoracic, and lumbar spine were performed which neuro-radiologists interpreted as C2-C3 moderate to severe right neural foraminal narrowing, C3-4 moderate to severe bilateral neural foraminal narrowing, and frank spinal cord compression at C4-C5 without definite abnormal intrinsic cord signal with moderate bilateral neural foraminal narrowing (Figures 1 and 2).

Neurology and internal medicine specialists were consulted, and after evaluation by these teams, the patient was deemed to have a myelopathic condition. Although the findings on MRI did reveal cord compression but were not conclusive of a myelopathic condition, given the severe progression of symptoms of motor weakness and sensory deficits, the decision was made that the patient would benefit from surgical intervention and the patient was prepared and optimized for surgery.

**Figure 3:**
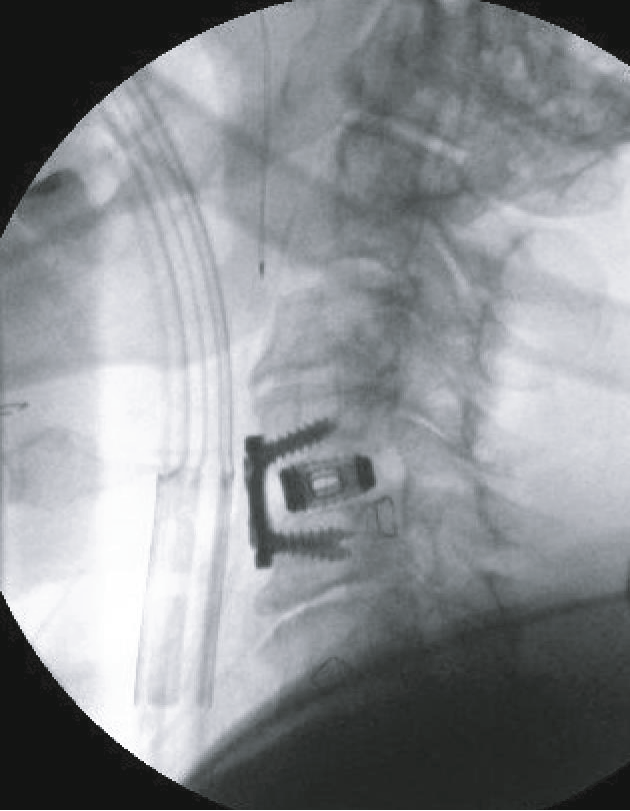
Lateral intraoperative radiograph of the cervical spine, status post-C4 and C5 anterior cervical discectomy and fusion.

**Figure 4:**
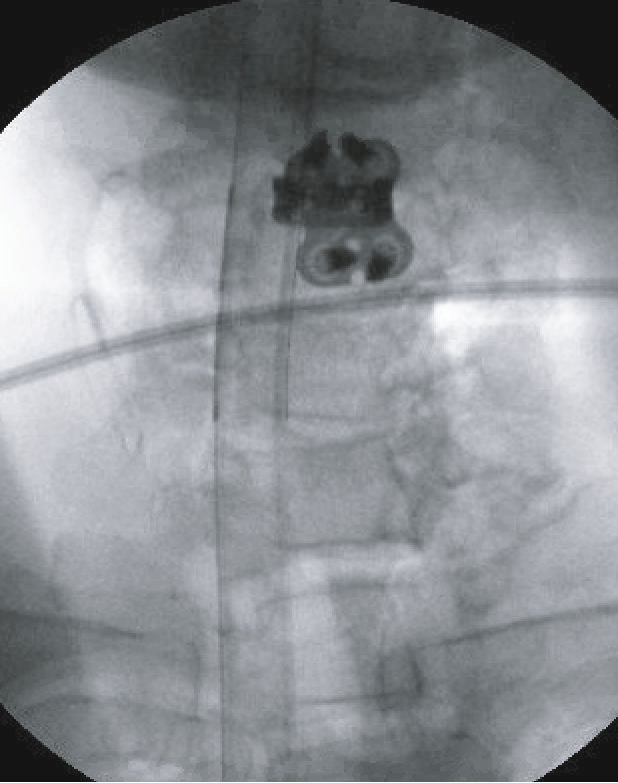
Anterior-posterior intraoperative radiograph of the cervical spine, status post-C4 and C5 anterior cervical discectomy and fusion.

One day after presentation to the hospital, the patient underwent an anterior cervical discectomy and fusion at the level of C4-C5 (Figures 3 and 4). The patient tolerated the procedure well, and there were no complications.

On postoperative day 1, clinical examination revealed mild improvement in motor strength in bilateral upper and lower extremities; however, the patient remained weak compared to baseline. Sensation improved and remained intact throughout the hospital stay. The patient was discharged from the hospital on postoperative day 7.

During a 3-month outpatient follow-up visit, clinical examination of the patient revealed improvement of neurologic symptoms. The patient was able to walk with assistance and a walker. Motor strength and sensation demonstrated improvement since the hospitalization with minimal pain. Clinical examination of the bilateral upper extremities revealed 4/5 motor strength in the deltoids, biceps, and triceps, proximally and distally, as well as an improved motor 3+/5 in grip strength. Examination of bilateral lower extremities revealed 4+/5 motor strength proximally in the hip flexors and knee extensors, with improvement of tibialis anterior, extensor hallucis longus, and peroneals to 3+/5 motor strength. Most importantly, the patient was able to perform basic activities of daily living, including self-feeding. Overall, the patient was ambulatory with a walker with significant improvement in functional status.

## 3. Discussion/Conclusion

In this case report, we outline an event in which a patient who experienced rapid neurological decline with myelopathic symptoms secondary to cervical spinal stenosis and radiographically confirmed myelomalacia was denied initial access to urgent clinical evaluation and treatment, in the setting of the COVID-19 pandemic. This event serves to shed light on the inherent issues surrounding access to care during a global and national health crisis. We describe a situation where a patient's clinical care and surgical treatment were delayed and urged independently by a surgeon due to the hesitancy of action by the system, caused by the fears and prejudices inherent during this pandemic that may have potentially caused irreversible damage and morbidity. We highlight here an issue that may have longer standing implications given the current state of the world and possible recurrences of similar global health crises in the future.

In reflection of the circumstances, several opportunities for education are available for the emergency medical services (EMS). While resource conservation during a medical catastrophe is sometimes necessary, acute neurological decline must be a consistent priority for the first responders. The EMS baseline assessment should include questions regarding changes in functional status, activities of daily life, bowel or bladder function, extremity use, and any fevers or chills associated with neurological decline. Whenever caring for a patient with a history of cognitive or neurological impairments, it is critical to consider the function of the patient in the weeks prior compared to the patient's present status with development of symptoms. If the patient's family is present, obtaining patient history from their perspective is invaluable. Similarly for patients living in extended care facilities, nursing staff and family members should be consulted when assessing baseline function and obtaining a detailed history. Through education of first-line responders, we can ensure vigilance of neurological symptoms and provide emergent care for at-risk patients who may otherwise experience a delay of care.

When EMS transports patients with neurological compromise, especially during a medical catastrophe, consideration must be given to which admitting facility the patient will be transported. During the pandemic, certain hospitals were not able to retain spine surgeons to operate at all hours, nor did they have the appropriate infrastructure to care for these types of patients. Certain facilities, which had limited high-quality imaging necessary for diagnosing myelopathic pathology, would not have been able to accurately diagnose nor care for a patient with neurological compromise. Those institutions without access to adequate critical care infrastructures, such as an ICU and the pertinent staff needed to care for these types of patients, would also not have been able to accommodate the needs of a postoperative spine patient. Our institution includes a Certified Center of Spine Excellence serving the greater New York Metropolitan Area and has the support staff to serve these acute patients. EMS needs to be aware of the services that specific medical centers are able to provide in order to continue to provide emergent care, especially in the midst of a pandemic.

It is also worthwhile to mention the beneficial utilization of telemedicine in the setting of a pandemic, where concerns and risk of transmission are high. In this case report, telehealth aided in the prompt recognition of a patient who needed emergent intervention to prevent further deterioration and permanent neurologic impairment. This case highlights the importance of the utility of teleconsultation, especially in these unique circumstances where direct in-person visits may not be accessible or feasible. Going forward, the development and use of this newer modality of patient evaluation may prove invaluable in medicine.

During this pandemic, there was a fear of exhaustion of resources and risk of contagion in hospitals. This was evident in the field triaging of patients deemed too critically ill to transport by EMS [16]. The use of EMS for another patient more likely to survive due to rapid intervention and transport was the justification in this scenario. In the end, society dictates which patients are appropriate to address with EMS; however, it must be considered that neurological decline may be just as grave as respiratory compromise during a pandemic. Therefore, there should still be a low threshold of suspicion for neurological compromise, in a patient exhibiting such symptoms to be brought in by EMS for further assessment. The cost of resource utilization and risks of spread are outweighed by the benefits of early intervention in the outcomes of patients with spinal pathology and subsequent neurological decline.

In the setting of surgical cancellations amidst a global pandemic, it is imperative that a scoring system is implemented that helps stratify the urgency of surgical management, so that similar situations to the one described do not become a common occurrence. Northwell Health uses a categorical system based on scores of 0-4, with a score of 4 reflecting a case with the highest urgency and need for surgical intervention in order to prevent secondary negative outcomes [10]. Although the patient in the case described in this report had a good overall outcome, there was still a delay in proper surgical intervention that may have resulted in permanent neurological damage and morbidity. Therefore, we strongly support the implementation of a multi-institutional guideline that also outlines the proper steps that must be taken at each level of patient care, including the first responders, surgeons, hospital administrators, and administrative chairs. Having a system that addresses the complicated nature of patient care during a pandemic or similar health crisis is of tantamount importance given the current state of our nation and the imperfect response to such a crisis in the age of such technological advancements in the modern era.
